# Is strength of handedness reliable over repeated testing? An examination of typical development and autism spectrum disorder

**DOI:** 10.3389/fpsyg.2015.00017

**Published:** 2015-02-03

**Authors:** Sara M. Scharoun, Pamela J. Bryden

**Affiliations:** ^1^Department of Kinesiology, University of WaterlooWaterloo, ON, Canada; ^2^Department of Kinesiology and Physical Education, Wilfrid Laurier UniversityWaterloo, ON, Canada

**Keywords:** handedness, hand preference, hand performance, children, autism spectrum disorder (ASD)

## Abstract

Despite a lack of agreement concerning the age at which adult-like patterns of handedness emerge, it is generally understood that hand preference presents early in life and development is variable. Young children (ages 3–5 years) are described as having weak hand preference; however, older children (ages 7–10 years) display stronger patterns. Here, strength of hand preference refers to reliable use of the preferred hand. In comparison to their typically developing (TD) peers, individuals with autism spectrum disorder (ASD) are described as having a weak hand preference. This study aimed to extend the literature to assess three measures of handedness (Waterloo Handedness Questionnaire – WHQ, Annett pegboard – AP, and WatHand Cabinet Test – WHCT) in two repeated sessions. The first research question aimed to delineate if the strength of hand use changes across testing sessions as a function of age in typical development. Right-handed children reported a reliable preference for the right hand on the WHQ, similar to adults. A marginally significant difference was revealed between 3- to 4- and 5- to 6-year-olds on the AP. This was attributed to weak lateralization in 3- to 4-year-olds, where the establishment of hand preference by age 6 leads to superior performance with the preferred hand in 5- to 6-year-olds. Finally, for the WHCT, 3- to 4-year-olds had the highest bimanual score, indicating use of the same hand to lift the cabinet door and retrieve an object. It is likely that the task was not motorically complex enough to drive preferred hand selection for older participants. The second research question sought to determine if there is difference between (TD) children and children with ASD. No differences were revealed; however, children with ASD did display variable AP performance, providing partial support for previous literature. Findings will be discussed in light of relevant literature.

## INTRODUCTION

Handedness is a multidimensional motor function which identifies the hand one prefers to use for a variety of unimanual tasks (i.e., *preference*) and the ability to perform more effectively with one hand (i.e., *performance*; Corey et al., 2001). Such dimensions enable handedness to be quantified according to *direction* and *degree*. Direction identifies whether an individual is left- or right-handed; whereas degree quantifies how strongly a person prefers one hand in comparison to the other both within a task and across time (Steenhuis and Bryden, 1989). A person with a strong hand preference reliably uses their preferred hand. A person with weak hand preference will typically use their preferred hand; however, they may switch to the non-preferred on occasion, thus displaying evident variability in hand selection. Finally a person with mixed or ambidextrous hand preference varies selection equally between both hands. Many studies have reported that approximately 90% of the human population is right handed. The proportion of right and left handers has remained reliable for approximately 5000 years (Coren and Porac, 1977). It is generally understood that left handers display less functional asymmetry than right handers (e.g., Springer and Deutsch, 1998; Yahagi and Kasai, 1999) and thus display an overall weak hand preference.

It has been suggested that preference for one hand emerges very early in life. Early lateralized motor behaviors (e.g., thumb sucking; Hepper et al., 1991), infant postural preferences (Coryell and Michel, 1978) and reaching, and grasping patterns (Marschik et al., 2008) are all thought to contribute to the development of handedness. From 6-months onward a preference for one hand can be detected (see Butterworth and Hopkins, 1993 for a review); however, hand use preference is both variable and malleable (Corbetta et al., 2006), such that different patterns of development are observed.

From early childhood to adolescence (i.e., 3- to 12-year-olds), consensus has not yet been reached regarding the age at which adult-like handedness is attained. It has been suggested that direction of preference is established at the age of 3. In comparison, degree increases between the ages of 3–7 years and more gradually until age 9 (Archer et al., 1988; Longoni and Orsini, 1988; McManus et al., 1988). From this, it is understood that assessment of hand preference is not reliable until age 4 (McManus, 2002). That said, others (e.g., Bryden et al., 2000a) have described that hand preference is not reliable until age 6, as 3- to 4-year-olds display variable patterns of handedness. Differences in developmental milestones of handedness are likely attributed to different ways of quantifying hand preference and performance abilities (see Scharoun and Bryden, 2014 for a review) as numerous tools are currently in use to quantify handedness. The following will speak to the development of handedness as assessed by means of: (1) measures of hand preference; (2) measures of hand performance; and (3) observational-based assessments of hand preference.

### MEASURES OF HAND PREFERENCE

Questionnaires are commonly used to identify the preferred hand for completing an activity (McManus and Bryden, 1992). These measures are based on a continuum from extreme left to extreme right, thus enabling quantification of both direction and, in some cases, degree (Steenhuis and Bryden, 1989) of hand preference. The Waterloo Handedness Questionnaire (WHQ) was used in the current investigation; however, this is one of numerous questionnaires in use. Although such questionnaires are not specifically designed for children, use is prevalent in the literature. For example, in the largest study to date, Carrothers (1947) had classroom teachers report on the handedness of 225,000 school children (grades 1–12) in Michigan. A general pattern of decline in left hand preference (i.e., an increase in right hand preference) was reported with age (Carrothers, 1947). Porac et al. (1980) also noted that the number of right handers increases with age. Two developmental hypotheses were presented to explain such trends: (1) environmental pressures toward right-handedness; and (2) neural development continuing into the third decade of life.

Previous research has also successfully utilized oral administration of questions as alterations to the administration of handedness questionnaires for children as young as 2 (Karapetsas and Vlachos, 1997; Cavill and Bryden, 2003). For example, Cavill and Bryden (2003) used the revised WHQ (20-item) to assess handedness in 2- to 24-year-olds. All age groups appeared right-handed and the distribution of hand preference did not change with age (Cavill and Bryden, 2003). Research to date thus outlines that right handers report a strong preference for the right hand over the course of development. In contrast, left handers display weak preference for the left hand which increases with age, albeit never reaching the degree of strength observed in right handers (e.g., Bryden et al., 2000b; Cavill and Bryden, 2003). Summarizing then, direction of hand preference appears to emerge at a relatively young age (Longoni and Orsini, 1988; McManus et al., 1988), whereas degree undergoes refinement with age.

### MEASURES OF HAND PERFORMANCE

Despite successful use of questionnaires with children, considering the subjective nature of their design, hand preference measures possess inherent limitations, and are not particularly reliable for use with children (Bryden et al., 2007). Finally, the large verbal and memory component limits use with children, especially those with developmental disabilities. Performance measures have thus been implemented to differentiate between right and left hand abilities on a particular task (McManus and Bryden, 1992). These measures include, but are not limited to, dot-filling tasks (Tapley and Bryden, 1985), peg-moving tasks (Matthews and Klove, 1964; Annett, 1970b), and manual aiming tasks (Roy and Elliott, 1989).

The current study used the Annett pegboard (AP), long established as a valid and reliable measure of hand performance, which times the movement of 10 doweling pegs (Annett, 1970b). Previous research with this method has revealed peg-moving time decreases with age (Kilshaw and Annett, 1983; Curt et al., 1992; Singh et al., 2001; Annett, 2002; Dellatolas et al., 2003). More specifically, between the ages of 3 and 6, a decrease in movement time by approximately 40% has been reported, alongside a decrease in variability of performance with age (Annett, 2002; Dellatolas et al., 2003). Some researchers have noted no change with age in the performance difference between the two hands (Kilshaw and Annett, 1983; Curt et al., 1992; Annett, 2002; Dellatolas et al., 2003), whereas others describe large performance differences in young children, which decrease with age (Roy et al., 2003; Bryden and Roy, 2005; Bryden et al., 2007). Performance differences have often been attributed to the development of the corpus callosum (e.g., Driesen and Raz, 1995).

### OBSERVATIONAL-BASED ASSESSMENTS OF HAND PREFERENCE

The inability to replicate findings in the literature highlight that despite the benefits of performance measures, similar to questionnaires, such measures possess their own limitations. To further elucidate the development of handedness observational-based have been implemented to assess children in a more natural environment (e.g., Kastner-Koller et al., 2007). For example, researchers have overcome these obstacles by means of asking children to perform each item listed on handedness questionnaires. Kilshaw and Annett (1983) observed the hand selected for the 12-item Annett (1970a) Handedness Questionnaire. Similar to other reports, no differences among the age groups were reported; however, younger children displayed weak hand use preferences, characterized by increased variability (i.e., switched between right-and left-hand) compared to older children (Kilshaw and Annett, 1983).

The WatHand Cabinet Test (WHCT; Bryden et al., 2000a), which was used in the current investigation, is another form of observational-based assessment of hand preference. This task enables a skilled score, consistency score, bimanual score, and total score to be computed. Due to minimum verbal requirements, the WHCT has been documented as an accurate means of assessing hand preference, in comparison to questionnaires (e.g., WHQ) and performance (e.g., peg-moving) measures. Bryden et al. (2007) have suggested it is an excellent tool for use with special populations.

Research with the WHCT has revealed young typically developing (TD) children (3- to 4-year-olds) are the least lateralized in comparison to older children and young adults, thus displaying weak hand preference tendencies. Furthermore, research with the WHCT has noted that hand preference is typically established at age 6 and the strength of preference increases with age. With age and maturation, older children (7- to 10-year-olds) display stronger, and therefore more reliable patterns of handedness. That said, Left handers generally display weak hand preference over the course of development, such that some young lefties use their non-preferred hand at least half of the time (Bryden et al., 2000b). All of that in consideration, the test–retest reliability of the WHCT has yet to be established; therefore, one aim of this study was to assess if strength of handedness changes over repeated testing sessions as a function of age.

### HAND PREFERENCE IN AUTISM SPECTRUM DISORDER

In comparison to their TD peers, an increased prevalence of left handedness has been reported in individuals with developmental disorders. Impaired left hemisphere functions causing a shift of localization to the right-hemisphere has been proposed (e.g., Geschwind and Behan, 1982). Autism spectrum disorder (ASD) is the most common form of severe developmental disability of childhood. Neural deficits are stereotypical of left hemisphere functions (i.e., language and comprehension skills) and the link between non-right handedness, left hemisphere dysfunction and ASD has become prevalent in the literature (Colby and Parkison, 1977; McCann, 1981; Gillberg, 1983; Neils and Aram, 1986; Soper et al., 1986; McManus and Bryden, 1992; Cornish and McManus, 1996).

Previous work with children with ASD has documented an obvious dissociation between hand preference and performance (McManus et al., 1992) as a result of patterns of lateralization that differ from TD children. For example, children with ASD performed better on the AP with the non-preferred hand, in comparison to the control, who displayed superior performance with the preferred hand (McManus et al., 1992). That said, Cornish and McManus (1996) have reported a decrease in left hand preference from 33% in younger children with ASD (ages 4–5) to 15% in older (ages 12–13) children with ASD. However, strength of preference was never fully comparable to TD children in their study. Cornish and McManus (1996) thus proposed children with ASD have a characteristic and individual pattern of handedness, described as non-right handedness. This idea has been repeatedly confirmed (Hauck and Dewey, 2001; Dane and Balci, 2007) using various preference, performance, and observational measures discussed previously. For example, Markoulakis et al. (2012) confirmed a greater proportion of left handers according to the WHCT, which contrasted self-declared hand preference. That said, reference to non-right-handedness in children with ASD typically refers to performance within a set of trials conducted in a single session. As such, this study aimed to extend the previous literature, by means of assessing handedness over repeated testing sessions in order to delineate if variability in strength of handedness is further exaggerated across time.

Overall, it is clear that young, TD children (3- to 5-year-olds) have weak hand preference tendencies, characterized by test–retest variability. With age and maturation, older TD children (7- to 10-year-olds) display an increase in strength of handedness. In other words, demonstrate more reliable use of the preferred hand. In comparison to their TD peers, children with ASD are described as having an increased frequency of non-right handedness. More specifically, increased rates of ambiguous and mixed-handedness have been documented. This study consists of an extension of previous work, as handedness was assessed in two repeated testing sessions to assess if hand preference tendencies, and performance differences between the two hands vary over time, as a function of age and between TD children and children with ASD.

This study used a cross-sectional approach to assess handedness, by means of preference (i.e., WHQ), performance (i.e., AP), and observational-based (i.e., WHCT) measures. Three different tools were implemented considering several factors may underlie handedness (e.g., Corey et al., 2001). Therefore it is clear to many researchers that a single test is not sufficient as numerous components of hand preference and performance must be considered. As outlined by De Agostini et al. (1992) “*the choice of the items used becomes crucial because this final classification is highly dependent on item choice…it should be stressed that the very young child may indeed manipulate an object with both hands not so much because his handedness is not yet established but rather because of factors that are independent of handedness*” (p. 54). Three distinct tools that correlate significantly as measures of handedness were thus selected (Bryden et al., 2000b; Brown et al., 2004). The AP was selected as a measure of hand performance. In comparison, the WHQ and WHCT were used to evaluate hand preference; the former through questionnaire and latter through observation. In other words, the WHCT can be considered an observational-based assessment of preference. Questionnaires are the most commonly and traditionally used assessments of hand preference (McManus and Bryden, 1992); however, considering problems with assessment in children, it has been suggested that measuring handedness through observation is an appropriate and effective alternative (e.g., Karapetsas and Vlachos, 1997). Additionally, these two measures of preference were selected, as the test–retest reliability of the WHCT has yet to be established. Thus, it was necessary to establish how reliable the measure was across time in relation to well established measures.

### HYPOTHESES

The specific research questions were as follows. First, does strength of handedness change over repeated testing sessions as a function of age? It was hypothesized that strength of handedness would be more reliably assessed over repeated testing sessions as a function of age. In other words, variability in performance would decrease as a function of age, such that younger children would display weak handedness tendencies, whereas older children would display stronger, and thus more reliable handedness tendencies. Secondly, is there a difference in strength of handedness when comparing TD children and children with ASD matched according to sex and comparable in chronological age? It was hypothesized that TD children would display stronger preference tendencies than children with ASD over repeated testing sessions.

## MATERIALS AND METHODS

### PARTICIPANTS

A cross-sectional approach was used to investigate handedness. Right-handed TD 3- to 12-year-old children (*n* = 76), a convenience sample, selected because of accessibility and proximity, of graduate, and undergraduate students from the researchers’ institution (*n* = 18) and a group of children with ASD (*n* = 13) participated in this study (see **Table 1**). The institution Research Ethics Board approved all recruitment and testing procedures. Informed consent was obtained.

**Table 1:** Participant demographics.

Group	*N*	Mean age (SD)	Male	Female
3- to 4-year-olds	11	3.64 (0.50)	5	6
5- to 6- year-olds	14	5.43 (0.51)	6	8
7- to 8-year-olds	21	7.43 (0.51)	10	11
9- to 10-year-olds	12	9.67 (0.49)	3	9
11- to 12-year-olds	18	11.22 (0.43)	8	10
Adults	18	21.44 (0.78)	10	8
Children with autism spectrum disorder (ASD)	13	8.38 (1.98)	8	5

Children with a formal diagnosis of ASD using DMS-IV-TR criteria (American Psychiatric Association, 2000) from a medical doctor were recruited to participate. This study was limited as IQ was not assessed; however, children were identified as high-functioning on the spectrum. After initial recruitment the autism spectrum quotient: children’s version (AQ-Child; Auyeung et al., 2008) was used as a means of quantifying autistic traits. A 50-item parent report questionnaire designed for 4- to 11-year-old children, the AQ-Child considers five areas associated with autism and the broader phenotype: social skills, attention switching, attention to detail, communication, and imagination. A four-point Likert scale is used to assess the degree to which parents agree/disagree with statements about their child (0: definitely agree; 1: slightly agree; 2: slightly disagree; and 3: definitely disagree). Items are reverse scored as necessary. Total AQ scores range from 0 (no autistic traits) to 150 (full endorsement on all items). A cut-off score of 76 has high sensitivity (95%) and specificity (95%); therefore, children with scores lower than 76 (*n* = 2) were excluded from analysis.

### PROCEDURES AND APPARATUS

Participants were seated at an age-appropriate table as they completed each task. Each participant was first asked which hand was used for writing (coloring for children) to denote self-report hand preference. Three distinct tools that correlate significantly as measures of handedness were used (Bryden et al., 2000b; Brown et al., 2004): (1) The WHQ, (2) The AP, and (3) The WHCT. To assess if reliable hand preference tendencies are displayed over repeated testing sessions, the entire battery of tests was completed on each of two separate days, with a minimum of 48 h between sessions.

#### Waterloo Handedness Questionnaire (WHQ)

The 32-item version of the questionnaire was used (Steenhuis et al., 1990). Each question permits five responses: “left always,” “left usually,” “uses both hands equally often,” “right usually,” and “right always.” A laterality quotient is computed by taking the difference between the total number of left and right hand responses [(right hand – left hand)/(right hand + left hand)] and multiplying the result by 100. It is expected, based on self-report hand preference, that left handers have a negative laterality quotient (i.e., left-hand preference) and right handers have a positive laterality quotient (i.e., right-hand preference).

Adult participants completed the questionnaire individually. The questionnaire was administered orally to TD children by reading each item aloud and explaining the item if necessary. It is important to note that previous research has successfully utilized oral administration of questions as alterations to the administration of handedness questionnaires for pre-school children (e.g., Karapetsas and Vlachos, 1997; Cavill and Bryden, 2003). That said, given the large verbal and memory components requirement, combined with the inability to distinguish how familiar children may be with particular tasks (e.g., which hand would you use to put a nut washer on a bolt; with which hand would you hold a needle when sewing?) the WHQ was not completed with 3- to 4-year-olds. In addition, children with ASD were either unable or unwilling to complete the questionnaire orally; therefore parents were asked to complete the questionnaire on behalf of their children on the first day of collection. As data was collected through different means, there was no means of direct comparison between TD children and children with ASD; therefore WHQ data was only used to confirm self-report hand preference.

#### Annett pegboard

In this task participants were required to pick up 10 doweling pegs, one at a time and place them into the empty holes as quickly as possible. Two trials were completed with the right and left hands. Starting hand was counterbalanced. The time to complete the task (i.e., hand performance) between touching the first peg to releasing the last was recorded using a stop-watch. If pegs were dropped, the trial was repeated. The average of the two trials for each hand was used for the purpose of analysis. Laterality quotients were the computed by taking the difference between left and right hand performance [(left hand - right hand)/(left hand + right hand)] and multiplying the result by 100. The size of the performance difference between the hands is thought to reflect the strength of hand preference (Provins and Magliaro, 1993). It is expected that left handers display negative laterality quotients and right handers display positive laterality quotient.

#### WatHand Cabinet Test

As outlined by Bryden et al. (2007), the WHCT

“was a cabinet 15.5^′′^× 12^′′^× 24^′′^. The cabinet was divided, in half, into compartments (one in the upper half and one in the lower half of the box). The top compartment was covered by a door that opened with a hand centered on the bottom edge of the door. The bottom compartment was not covered. The cabinet included two cup hooks centered on the left-hand side (while facing the front) of the cabinet, three inches apart, one above the other. A screw was centered on the right-hand side of the box, a Velcro bull’s eye target and ball were located on the top at the back of the cabinet, and a small padlock hung from a hook that was centered on the door located at the front of the cabinet” (p. 831).

Bryden et al. (2007) procedures were followed: “lifting the cabinet door a total of four times, using a toy hammer, placing rings on hooks, tossing a ball to a target, opening a lock with a key, using a screwdriver, pushing small buttons on a gadget, picking up a candy dispenser that was behind the cabinet door” (p. 831). For the purpose of analyses, four sub-scores were computed. The *total score* considered performance of all unimanual tasks; whereas the *skilled score* considered seven tasks that required manual dexterity (i.e., use a toy hammer, place a washer on a hook, toss a ball to a target, open a lock with a key, use a screwdriver, push small buttons on a gadget, use a crayon). These scores were calculated with a laterality quotient by taking the difference between the total number of left and right hand responses [(right hand – left hand)/(right hand + left hand)] and multiplying the result by 100. A *consistency score* was also computed by averaging right hand performance of the four door lift tasks (scored 0, 1, 2, 3, or 4 out of 4; Bryden et al., 2007). Finally, a *bimanual score* was by recording whether the hand used to open the cabinet door was the same to retrieve the candy dispenser. A score of 1 was given if opposite hands were used, whereas a score of 2 was given if participants used the same hand for both elements of the task, for a total possible eight points.

## RESULTS

### HANDEDNESS IN TYPICAL DEVELOPMENT

The first stage of analysis examined the overall relationship between scores obtained by right-handers in the first and second session. This was done to assess how reliable the measures were across time. Correlation analysis revealed a significant positive relationship between laterality quotients computed from the WHQ, *r* = 0.84, *p* < 0.01, and AP, *r* = 0.44, *p* < 0.01. For the WHCT, significant positive relationships were revealed for the total (*r* = 0.55, *p* < 0.01), skilled (*r* = 0.49, *p* < 0.01), consistency (*r* = 0.49, *p* < 0.01), and bimanual (*r* = 0.72, *p* < 0.01) scores. For subsequent analysis, participants were split into six separate age groups (3- to 4-year-olds, 5- to 6-year-olds, 7- to 8-year-olds, 9- to 10-year-olds, 11- to 12-year-olds, and adults). The following will outline results derived from the WHQ, AP, and WatHand Cabinet tasks.

#### Waterloo Handedness Questionnaire

As 3- to 4-year-olds did not complete the WHQ, analysis was limited to 5- to 12-year-old children and adults. An analysis of variance (ANOVA) test was used to analyze laterality quotients computed form the WHQ as a factor of Age (x5: 5- to 6-, 7- to 8-, 9- to 10-, 11- to 12-year-olds, adults) and Session (x2: first session, second session). There was a main effect of Session [*F*(1,78) = 9.933, *p* = 0.002, η^2^ = 0.113]. Laterality quotients were more positive in the second session (*M* = 79.15, SD = 30.15) in comparison to the first (*M* = 73.00, SD = 31.49). Neither a main effect of Age, nor a Session × Age interaction was revealed.

#### Annett pegboard

**FIGURE 1:**
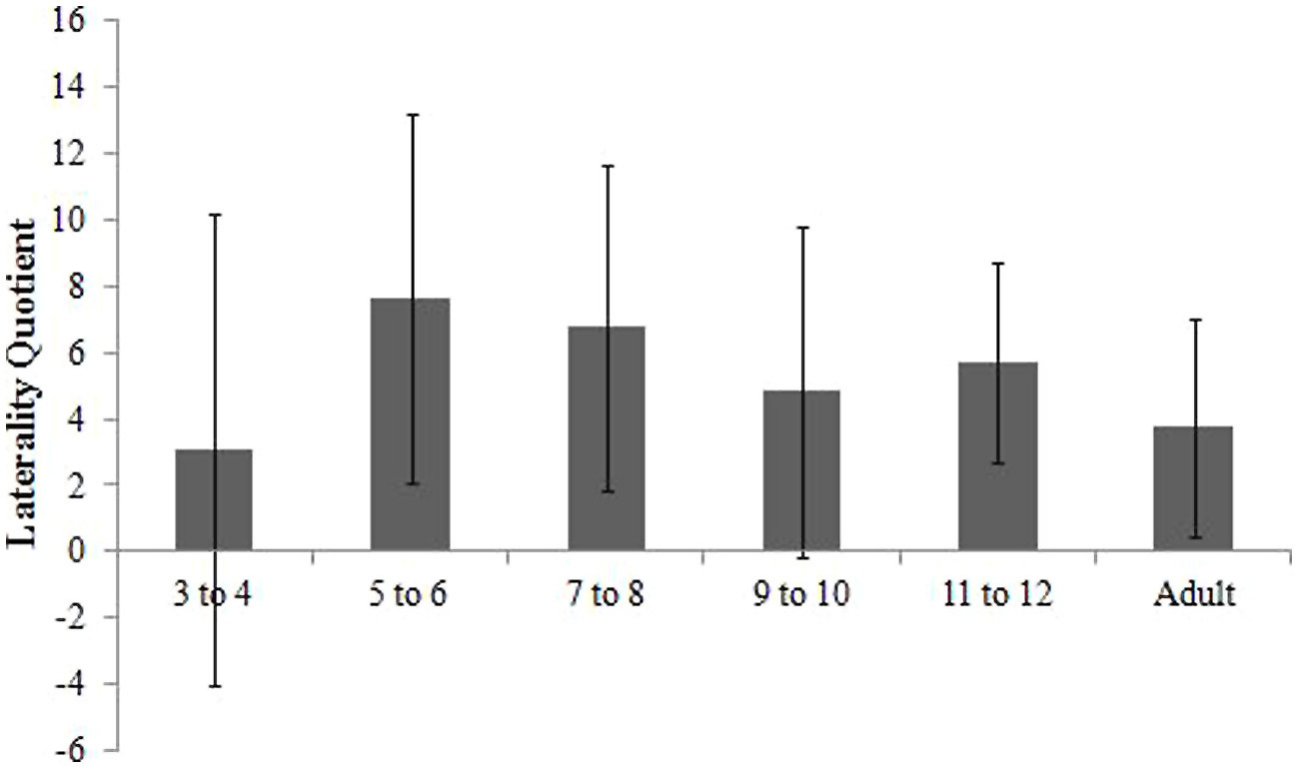
**Laterality quotients computed from typically-developing participants’ Annett Pegboard Task**.

All participants completed the AP task. An ANOVA was used to assess laterality quotients computed (see **Figure 1**), as a function of Age (x6: 3- to 4-, 5- to 6-, 7- to 8-, 9- to 10-, 11- to 12-year-olds, adults), and Session (x2: first session, second session). There was a main effect of session [*F*(1,88) = 3.971, *p* = 0.049, η^2^ = 0.043]. Laterality quotients were more positive (i.e., greater difference between the two hands favoring the right-hand] in the first session (*M* = 5.91, SD = 4.79) compared to the second (*M* = 4.73, SE = 0.54). There was also a main effect of age [*F*(5,88) = 2.752, *p* = 0.023, η^2^ = 0.135]. *Post hoc* tests using a Bonferroni correction for multiple comparisons displayed the difference between 5- to 6-year-olds and 3- to 4-year-olds was not far from reaching statistical significance (*p* = 0.090), such that 5- to 6-year-olds displayed more positive laterality quotients (i.e., greater performance difference between the two hands favoring the right-hand) compared to 3- to 4-year-olds. A Session × Age interaction was not revealed.

#### WatHand Cabinet Test

**FIGURE 2:**
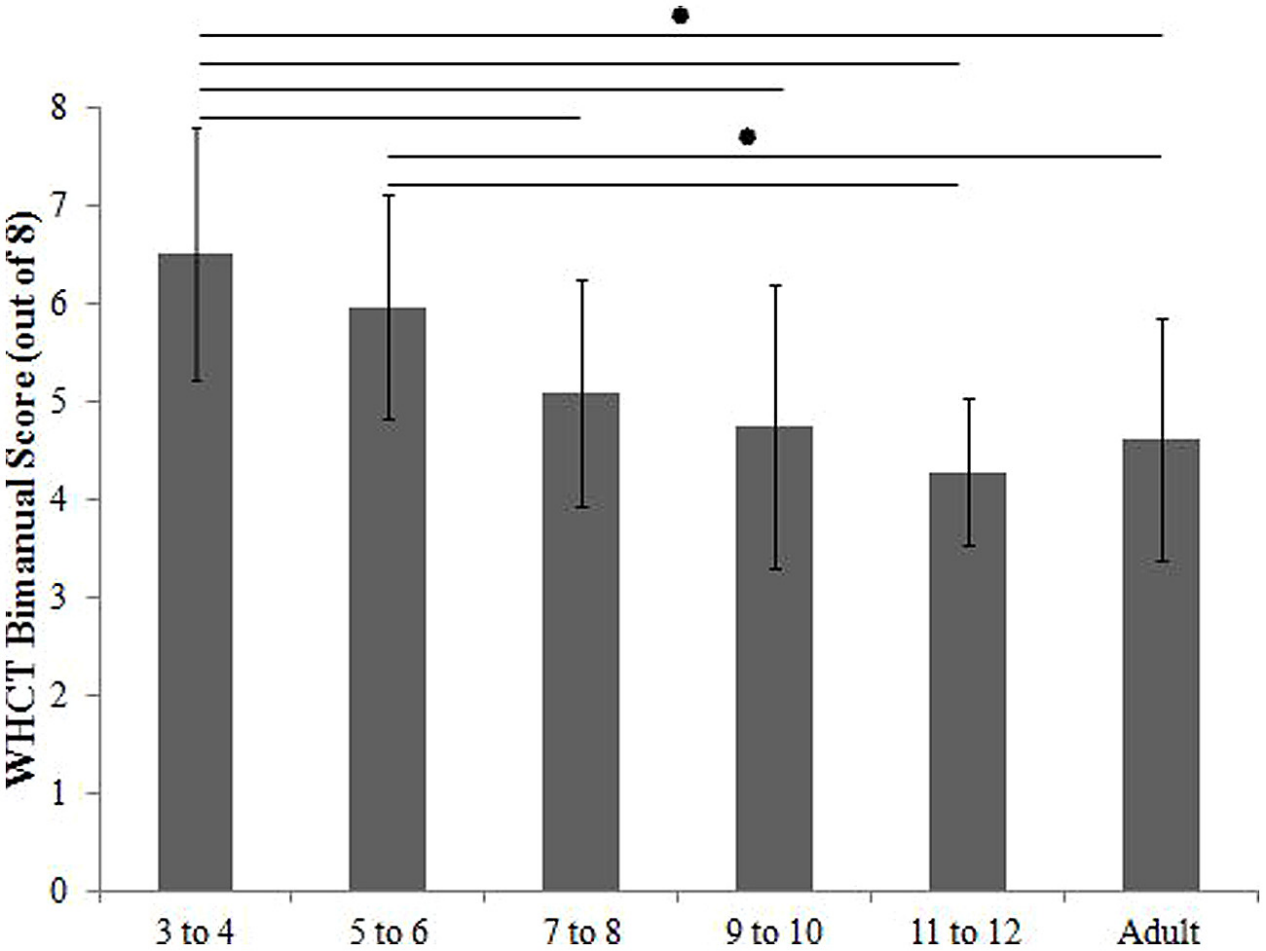
**Typically-developing participants’ bimanual scores computed form the WatHand Cabinet Test**.

Separate ANOVAs were performed for each of the four sub scores (total, skilled, consistency, and bimanual scores), as a function of Age (x6: 3- to 4-, 5- to 6-, 7- to 8-, 9- to 10-, 11- to 12-year-olds, adults), and Session (x2: first session, second session). No significant main effects or interactions were revealed for the total, skilled, and consistency score (*p* < 0.05). For the bimanual score (see **Figure 2**), there was a main effect of age [*F*(5,88) = 8.956, *p* < 0.001, η^2^ = 0.337]. *Post hoc* tests using a Bonferroni correction for multiple comparisons displayed 3- to 4-year-olds had significantly higher scores than 7- to 12-year-olds and adults. There was no difference between 3- to 4- and 5- to 6-year-olds. The 5- to 6-year-olds had significantly higher scores than 11- to 12-year-olds and adults. This indicates that 3- to 4-year-olds were more likely than 7- to 12-year-olds and adults to lift the cabinet door and retrieve the object from within the cabinet with the same hand; whereas, 5- to 6-year-olds were more likely than 11- to 12-year-olds and adults to lift the cabinet door and retrieve the object from within the cabinet with the same hand.

### CHILDREN WITH ASD AND THEIR TYPICALLY DEVELOPING PEERS

Thirteen children with ASD between the ages of 5 and 11 participated in this portion of the study. Two children did not complete the entire battery of tests on the second day of testing; therefore they were excluded from analysis. After data collection, two additional children were excluded from analysis because their AQ-Child scores were below the cut off of 76. The nine children with ASD (six male, three female, *M*_age_ = 8.11, SD = 1.96) remaining had a range of AQ total scores from 76 to 121 (*M*_score_ = 98.67, SD = 13.11). The WHQ identified one female participant as left handed (score = -100), whereas the remaining participants were right handed (*M*_score_ = 97.60, SD = 4.70).

Considering differences in the ratio of male to female and right to left participants in both groups (i.e., children with ASD and TD children), only right-handed male children were included, to match according to sex and comparable in chronological age. As such, analysis included six male children with ASD (*M*_age_ = 7.50, SD = 3.07; *M*_AQ-score_ = 101.67, SD = 10.13) and 24 TD children from the first sample (*M*_age_ = 8.00, SD = 1.98; **Table 2**). As mentioned previously, children with ASD were either unable or unwilling to complete the WHQ orally; therefore parents were asked to complete the questionnaire on behalf of their children on the first day of collection. As data was collected through different means, there was no way of comparing TD children and children with ASD directly; therefore WHQ data was only used to confirm self-report hand preference.

**Table 2:** Participant demographics – comparison between typically-developing (TD) children and children with ASD (ONLY RH male children included in analysis).

Group	*N*	Mean age (SD)
Children with ASD	6	7.50 (3.07)
TD Children	24	8.00 (1.98)

The first stage of analysis examined the overall relationship between scores obtained by children with ASD in the first and second session. This was done to assess how reliable the measures were across time in this sample of children. Correlation analysis revealed a non-significant negative correlation between laterality quotients computed from the AP, *r* = -0.62, *p* = 0.19. For the WHCT, significant positive relationships were revealed for the total (*r* = 0.89, *p* < 0.05) and consistency (*r* = 0.89, *p* < 0.05) scores. Skilled (*r* = 0.77, *p* = 0.07), and bimanual (*r* = 0.55, *p* = 0.26) scores were not correlated. Subsequent analyses compared the performance of children with ASD and their TD peers. The following section will outline the comparison between male right-handed TD children and children with ASD on the AP and WHCT. To help explain the high standard deviations, minimum and maximum scores are listed in **Table 3**.

**Table 3:** Minimum and maximum scores for TD children and children with ASD.

	TD Children		Children with ASD	
	Minimum	Maximum	Minimum	Maximum
**Annett pegboard**
Session 1	-5.47	15.26	5.31	13.21
Session 2	-4.10	17.58	-5.79	6.85
**WHCT total**
Session 1	-20.00	100.00	40.00	100.00
Session 2	-40.00	100.00	60.00	100.00
**WHCT skilled**
Session 1	33.33	100.00	42.86	100.00
Session 2	14.29	100.00	71.43	100.00
**WHCT consistency**
Session 1	0.00	4.00	3.00	2.00
Session 2	0.00	4.00	4.00	4.00
**WHCT bimanual**
Session 1	4.00	8.00	4.00	8.00
Session 2	4.00	8.00	4.00	8.00

#### Annett pegboard

**FIGURE 3:**
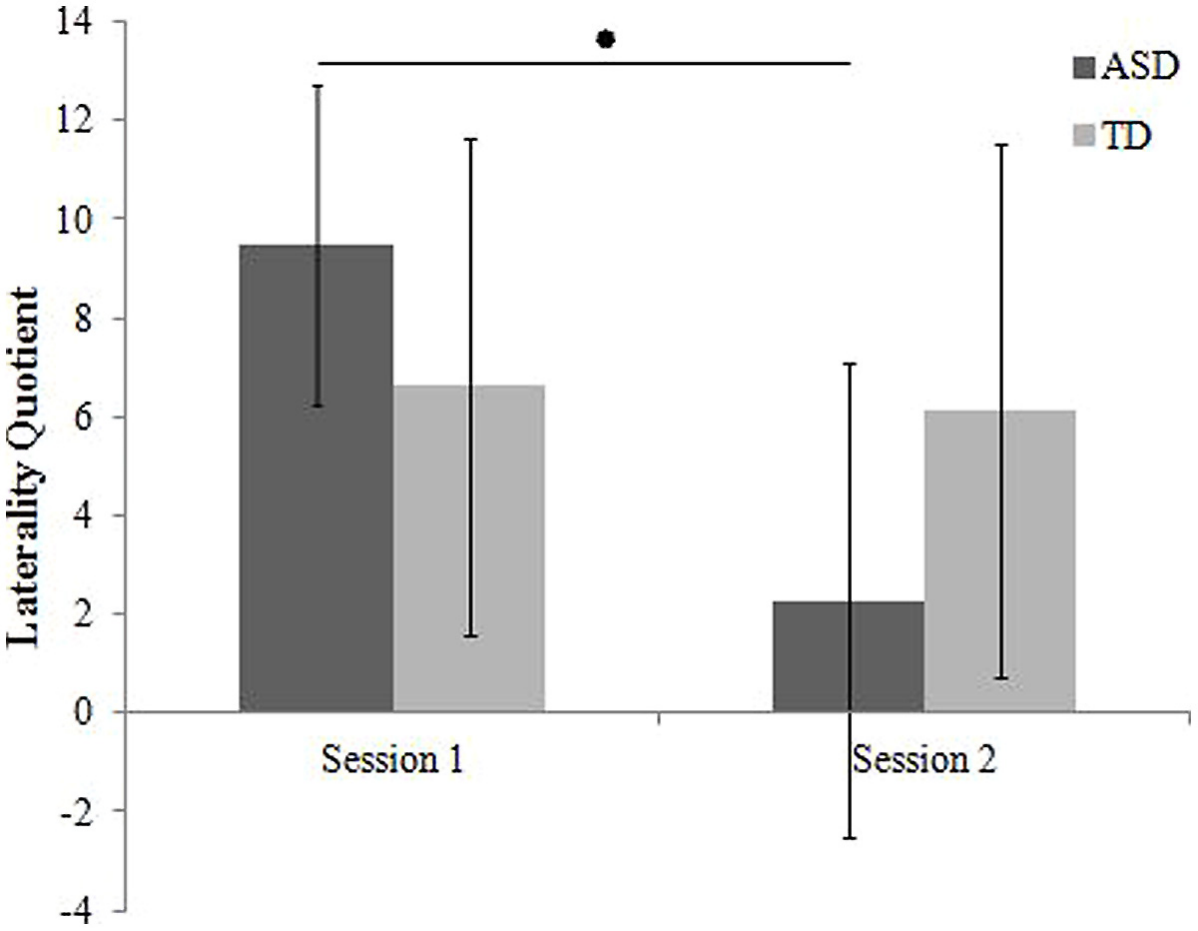
**Laterality quotients computed from the Annett Pegboard**.

An ANOVA was used to assess laterality quotients computed from the AP (see **Figure 3**), as a function of Group (x2: TD children, children with ASD), and Session (x2: first session, second session). There was a main effect of Session [*F*(1,28) = 8.686, *p* = 0.006, η^2^ = 0.237] and a Session × group interaction [*F*(1,28) = 6.632, *p* = 0.016, η^2^ = 0.191]. Laterality quotients were more positive in the first session compared to the second; however, the Session × group interaction revealed this was due to children with ASD, who had more positive laterality quotients (i.e., greater difference between the two hands favoring the right-hand) in the first session. There was no main effect of group.

#### WatHand Cabinet Test

Separate ANOVAs were performed for each of the four sub scores (total, skilled, consistency, and bimanual scores), as a function of Group (x2: TD children, children with ASD), and Session (x2: first session, second session). No significant effects or interactions were revealed in analyses for the total, skilled, or consistency scores (*p* > 0.05). For the bimanual score, there was a main effect of Session [*F*(1,28) = 6.760, *p* = 0.015, η^2^ = 0.194]. Bimanual scores were greater in the first session (*M* = 5.229, SD = 1.416) compared to the second (*M* = 4.688, SD = 1.095). No main effect of group or Session × group interaction emerged.

## DISCUSSION

It is generally understood that young, TD children (3- to 4-year-olds) display weak hand preference tendencies. Furthermore, it is argued that hand preference is established at age 6 and strength improves with age (see Scharoun and Bryden, 2014 for a review). At the age of 6 children are learning to write; therefore improved writing skills may explain an increase in the strength of hand preference with age (e.g., McManus et al., 1988). Observing children with ASD in comparison to their TD peers, variable hand selection strategies have been noted, such that children with ASD are described as having ‘mixed-preference’ or an overall pattern of non-right handedness (e.g., Cornish and McManus, 1996). Clearly *strength* of handedness is a topic that is continuously discussed in the handedness literature.

With that in mind, the current study addressed two specific research questions. First, does strength of handedness change over repeated testing sessions as a function of age? It was hypothesized that strength of handedness would be more reliably assessed over repeated testing sessions as a function of age. In other words, variability in performance would decrease as a function of age, such that younger children would display weak handedness tendencies, whereas older children would display stronger, and thus more reliably handedness tendencies. Secondly, is there a difference in strength of handedness when comparing TD children and children with ASD matched according to sex and comparable in chronological age? It was hypothesized that TD children would display stronger preference tendencies than children with ASD over repeated testing sessions. The following will discuss each research question and hypothesis in turn.

### HANDEDNESS IN TYPICAL DEVELOPMENT

#### The Waterloo Handedness Questionnaire

Research to date outlines that right handed children report a reliable preference for the right hand, similar to adults (e.g., Bryden et al., 1991). In line with previous findings, an overall right hand preference was observed (Steenhuis and Bryden, 1989; Steenhuis et al., 1990). Interestingly, regardless of age, participants demonstrated a significantly stronger right hand preference during the second testing session. This was likely due to familiarity with the questions. Anecdotally, participants took longer to complete the questionnaire in the first session. It can be presumed that more thought was being put into answers.

#### The Annett pegboard

Previous research has displayed variable results with respect to performance on the AP. Some suggest that asymmetries do not change as a function of age (Kilshaw and Annett, 1983; Curt et al., 1992; Annett, 2002; Dellatolas et al., 2003). However, others (Roy et al., 2003; Bryden et al., 2007) have noted children display greater performance differences between the hands than adults. Results of the current study do not agree with either hypothesis. The difference between 5- to 6-year-olds and 3- to 4-year-olds was not far from reaching statistical significance (*p* = 0.090). The 5- to 6-year-olds displayed more positive laterality quotients (i.e., greater performance difference between the two hands favoring the right-hand) compared to 3- to 4-year-olds. No other differences between age groups were noted. These results provide partial support for Annett (2002), who described that, “differences are slightly larger in young than older children but this is a function of the rapid rates of growth in the early years” (p. 552). This does not explain the performance of 3- to 4-year-olds.

Bryden et al. (2000a) have suggested that young TD children (3- to 4-year-olds) are the least lateralized and therefore display minimal performance differences between the hands. By age 6, however, “handedness has been firmly entrenched” (Bryden et al., 2000b, p. 64). Pryde et al. (2000) have proposed that older children (i.e., 6- to 10-year-olds) “tend to think in concrete, inflexible terms and are undergoing a period of motor skill refinement” (p. 374). As such, older children are described as showing an overuse of the preferred hand. In other words, reliably use their preferred hand, regardless of task, or region of space. In line with this idea, it is likely that, due to weak hand preference 3- to 4-year-olds displayed small performance differences between the two hands (Annett, 2002; Dellatolas et al., 2003). As hand preference is typically established at age 6 (Bryden et al., 2000b), this likely explains why 5- to 6-year-olds displayed large performance differences between the two hands, in favor of the preferred hand. De Agostini et al. (1992) observed a greater proportion of mixed-handed children at age 3 than at age 6. They explained that “the decreasing percentage of mixed-handed children with age contributed to the increase of full right-handed children” (p. 53). According to Fennell et al. (1983), hand preference at age 5 predicted handedness for 97% of right-handers at age 11. It is thus likely that a right-hand was established in the 5- to 6-year-olds in this study, whereas 3- to 4-year-olds displayed more of a mixed-preference.

#### The WatHand Cabinet Test

Performance measures, like the AP, have inherent limitations, as they only measure one aspect of handedness – in this case, speed. The WHCT, an observational-based assessment, has been shown to be the most accurate predictor of hand preference (Brown et al., 2004), especially for use with children (Bryden et al., 2007). That said, previous studies have based their conclusions on a single testing session (e.g., Brown et al., 2004; Bryden et al., 2007); whereas the current study was completed on two repeated testing days, in order to measure if handedness can be reliably assessed with this task. Paralleling previous studies (e.g., Bryden et al., 2007), four sub-scores were computed: a total score, skilled score, consistency score, and bimanual score. No significant main effects or interactions were revealed for the total, skilled or consistency score with respect to age or session. This indicates right handed children reliably display a preference for one hand within these tasks. For the bimanual score, 3- to 4-year-olds had the highest scores. Thus, they were more likely to lift the cabinet door and retrieve the object from within the cabinet with the same hand, whereas older children and adults used. This result is in line with a previous report from Bryden et al. (2007) who noted younger children showed a stronger preference for the preferred hand. However, in Bryden et al.’s (2007) study, this was true for 3- to 9-year-old children, where this was limited to 3- to 4-year-olds in the current study. Bryden et al. (2007) suggest that “it may be that the two tasks (opening a door and picking up an object) were not considered motorically complex enough to drive the selection of the preferred hand for older individuals” (p. 840) and that “experience could have decreased the older participants reliance on the preferred hand” (p. 840–841). That said, it may also be a function of corpus callosum maturation. With age, there is an evident transition from a unimanual strategy to a bimanual strategy (e.g., Fagard and Corroyer, 2003).

### Handedness in ASD

The second objective of this study was to investigate whether a group of children with ASD demonstrate the same strength of handedness as their TD peers, as variable hand preference tendencies have been reported within performance of a single task (e.g., McManus et al., 1992; Cornish and McManus, 1996; Markoulakis et al., 2012). Based on previous reports in the literature, it was hypothesized that children with ASD would demonstrate variable hand use strategies in comparison to their TD counterparts. In partial agreement with previous findings, the current study did observe some evidence of variable hand use tendencies in children with ASD (e.g., McManus et al., 1992; Cornish and McManus, 1996; Markoulakis et al., 2012), although this was limited.

#### The Annett pegboard

In the current study, there was no statistically significant difference between children with ASD and their TD counterparts. These results are in line with previous reports in the literature which indicate that performance differences between TD children and children with ASD typically subside when measuring the difference between the two hands using laterality quotients (Cornish and McManus, 1996). This study adds to the literature, suggesting that this extends to assessment over repeated sessions. That said, correlation analysis revealed a non-significant negative correlation between AP scores in the first and second session. This suggests that, as a group, children with ASD do display more variable handedness.

#### The WatHand Cabinet Test

Results of this study revealed no differences between children with ASD and their TD peers in any of the sub-scores of the WHCT (i.e., total score, skilled score, consistency score, and bimanual score). These findings opposed those found recently in the literature by Markoulakis et al. (2012), who noted variable hand-use strategies in one testing session.

## SUMMARY AND CONCLUSION

The first research question asked if strength of handedness changes over repeated testing sessions as a function of age. With respect to hand preference, results from the WHQ contrasted the hypothesis, but were in line with previous reports which note that, similar to adults, children report a reliable preference for the right hand (Bryden et al., 1991). That said, significantly stronger right hand preference was seen in the second session, which begs the question of how familiarity with the questions influences participant response. Next, the WHCT, a performance-based assessment of preference, revealed that 3- to 4-year-olds had higher bimanual scores than all other age groups. This was again in contrast with the hypothesis, but in line with Bryden et al. (2007) who noted that the task may not be complex enough to drive preferred hand selection in older participants. Adding to the literature, there were no differences in hand use preferences between sessions; providing evidence that the WHCT is a reliable measure. Finally, with respect to the AP, the measure of performance used in the study, a marginally significant difference was revealed between 3- to 4- and 5- to 6-year-olds, where 5- to 6-year-olds displayed greater performance differences between the two hands favoring the right-hand. As 3- to 4-year-olds are known to display weak hand preference tendencies, and hand preference is known to be entrenched by the age of 6 (Bryden et al., 2000a), this result is also in line with previous reports. Summarizing then, results of this study provide additional evidence to support the notion that 3- to 4-year-olds show weaker handedness in comparison to older children and adults. Thus it is clear that, despite weak tendencies within a session, children in this age group reliably display a weak pattern of handedness from one session to the next.

The second research question asked if a difference in strength of handedness is evident when comparing TD children and children with ASD matched according to sex and comparable in chronological age. Results were in partial support of the the hypothesis, such that there was no difference between TD children and children with ASD within each of the tasks, or between repeated testing sessions; however, performance of the AP revealed children with ASD displayed more variable handedness, exemplified by more positive laterality quotients in the first session, compared to the second. This was in contrast to TD children who demonstrated reliable strength in handedness.

Results of this study must be interpreted in light of limitations. In particular, this study included a small group of children with ASD, in comparison to a large group of TD controls. Comparison was limited to self-report right-handed male children matched according to sex and comparable in chronological age. It is thus possible that differences may be attributed to children’s IQ even though children were identified as high-functioning prior to their participation in this study. In conclusion, results of this study identify the need for continued examination of hand preference and motor skills in children with ASD. It has been argued that motor deficits are a cardinal feature of ASD (Fournier et al., 2010), are more common than in TD individuals (Matson and Kozlowski, 2011) and may significantly affect social development and overall quality of life (Gowen and Hamilton, 2013). However, variable performance is commonly reported and the etiology remains unclear (Gowen and Hamilton, 2013). Clearly, future research is warranted.

## Conflict of Interest Statement

The authors declare that the research was conducted in the absence of any commercial or financial relationships that could be construed as a potential conflict of interest.
